# The Clinical Impact of Rapid, Direct MALDI-ToF Identification of Bacteria from Positive Blood Cultures

**DOI:** 10.1371/journal.pone.0169332

**Published:** 2016-12-30

**Authors:** Kathryn French, Jason Evans, Hannah Tanner, Savita Gossain, Abid Hussain

**Affiliations:** Public Health England, Public Health Laboratory Birmingham, Heart of England NHS Foundation Trust, Birmingham, United Kingdom; Animal and Plant Health Agency, UNITED KINGDOM

## Abstract

**Background:**

Faster identification of bacterial isolates from blood cultures can enable earlier clinical intervention for patients with sepsis. We evaluated the clinical impact of direct identification of micro-organisms from positive blood cultures using MALDI-ToF.

**Method:**

Positive blood cultures with organisms seen on Gram stain were included over a four week period. For each patient case, comparison was made between the clinical advice given on day one with only a Gram stain result, and the follow up advice given on day two with the benefit of organism identification. Culture results were then compared with direct MALDI-ToF identification.

**Results:**

For 73 of 115 cases (63.5%), direct organism identification was obtained by MALDI-ToF. Of those 73, 70 (95.5%) had a result concordant with that of the plate culture. In 28 of the 115 cases (24.3%) direct MALDI-ToF identification on day one would have had a clear clinical benefit. In 11 cases it would have helped to identify the potential source of bacteraemia. In 11 cases it would have indicated a different antibiotic regimen on day one, with five patients receiving appropriate antibiotics 24 hours earlier. For 14 cases the blood culture isolate could have been designated as unlikely to be clinically significant.

**Conclusion:**

We have demonstrated that organism identification on day one of blood culture positivity can have a direct clinical impact. Faster identification using MALDI-ToF assists the clinician in assessing the significance of a blood culture isolate on day one. It can allow earlier appropriate choice of antimicrobial agent, even in the absence of susceptibility testing, and help narrow down the potential source of infection providing a focus for further investigation in a more timely way than conventional techniques alone.

## Introduction

Bacteraemia in the acutely unwell patient carries a significant morbidity and mortality. Prompt treatment with appropriate antimicrobial agents, along with supportive care, improves patient outcome [1]. However, conventional culture techniques are time-consuming with results often only available 48 hours after the patient has presented with an acute illness. Prompt identification of microorganisms grown in blood cultures has the potential to enable earlier targeted clinical intervention for patients with sepsis. Matrix assisted laser desorption ionization time-of-flight (MALDI ToF) mass spectrometry has been established as a rapid method for identification of cultured bacterial isolates [2]. Use of this technology has been extended to identification of bacteria directly from positive blood culture broths with some success [3–6]. Rates for correct identification to species level are reported as between 71.2% [7] and 85.5% [4], with MALDI-ToF consistently demonstrating greater success in identification of Gram negative organisms when compared with Gram positive organisms [3,4]. Lagacé-Weins *et al* [8] report that using MALDI-ToF on positive blood culture broths significantly reduces time to organism identification with a mean time reduction of 34.3 hours. Similar results have been reported by Schneiderhan *et al* [9] who report a mean reduction in time to organism identification of 58.7 hours, although the authors acknowledge this is dependent upon workflow and continuous blood culture incubation and processing. However, it is unclear the extent to which earlier species identification directly from blood culture broths can impact on clinical decision making. Clerc *et al* [10] report a series of 202 cases of Gram negative bacteraemia with 35.1% of cases having changes made to antimicrobial therapy as a result of MALDI-ToF identification, as opposed to 20.8% of cases with Gram stain result only. Similarly, Vlek *et al* [11] describe an increase in the proportion of patients on adequate antimicrobial therapy within 24 hours of a positive blood culture when using MALDI-ToF identification directly from broths. However, clinical management encompasses more than choice of antimicrobial agent alone.

We assess the impact of direct MALDI-ToF identification of blood culture isolates on a range of clinical decisions including source investigation, infection control management, and antimicrobial therapy choices.

## Materials and Methods

### Blood culture

Blood was cultured in the Public Health Laboratory Birmingham using the bioMérieux BacT/ALERT^®^ 3D system. Blood cultures used in this study were received in aerobic and anaerobic bottles containing no charcoal which are designed to take up to 10ml of blood. Within laboratory working hours (7am to 7pm), bottles were removed from the incubator when microbial growth in the culture bottles was indicated. A Gram stain was performed directly on an aliquot of culture. Based on the morphology of the organisms seen, culture plates were inoculated, incubated for 18-48hrs and examined daily for microbial growth. Identification of cultured isolates was performed using the Bruker MALDI Biotyper system for MALDI-ToF or BioMerieux VITEK^®^2 ID system.

### Direct MALDI-ToF

Briefly, 1 ml of each positive blood culture (with organisms seen on Gram stain) was mixed with 200 μl of 10% sodium dodecyl sulphate (SDS) solution, briefly vortex mixed and incubated at room temperature for 5 min then centrifuged for 1 min at 13,000 g. The supernatant was removed and each pellet re-suspended in 1 ml mass spectrometry grade water then centrifuged for 1 min at 13,000 g. Supernatant was removed and each sample was re-suspended in 300 μl mass spectrometry grade water and 900 μl 100% ethanol followed by centrifugation for 2 minutes at 13,000 g. The supernatant was then removed and each sample centrifuged again for 2 minutes at 13,000 g. Residual ethanol was then removed and the pellets dried for 10 minutes at room temperature. Ethanol/formic acid extraction was then carried out. One microliter of each extracted supernatant was spotted in duplicate onto MALDI-ToF target plates, dried and 1 μl α-Cyano-4-hydroxycinnamic acid (HCCA) matrix solution was added and dried. Genus and species identification was then obtained using a Bruker MALDI Biotyper system.

### Clinical data collection

Clinical data was collected over four weeks in December 2013. All positive blood cultures with organisms seen on Gram stain from patients over 18 years of age were included, and each individual patient case examined. For each case, the following data were collected on day one with the Gram stain result, and again on day two when the organism identification was known: likely clinical significance of the positive blood culture isolate; likely source or focus of infection; recommended clinical or laboratory investigations; advice regarding antimicrobial chemotherapy; recommended infection control interventions. A comparison was made between advice given on day one with only a Gram stain result, and the follow up advice given on day two with the benefit of organism identification. Changes in clinical advice of the above categories were recorded.

### Comparison of culture identification with direct MALDI-ToF identification

Direct MALDI-ToF identification was performed in parallel with conventional culture for all included cases. Identification generated from the direct MALDI-ToF method was not disclosed to the medical microbiologist until after culture results were available to avoid contamination of clinical decision making with a non-validated result. In order to correctly attribute any patient management changes to an earlier organism identification it was necessary to establish that the direct MALDI-ToF method would correctly provide that identification. Day two conventional culture identification results were therefore compared with the results generated through direct MALDI-ToF identification from blood culture bottles. Cases where MALDI-ToF gave an organism identification were considered as having the potential to change clinical management on day one. All cases were reviewed by an independent second assessor. Incidents of disagreement between the assessors were discussed further until consensus was reached.

This project was approved by internal ethical review by the Public Health England Research Support and Governance Office. Consent from patients was not sought as the study was a comparison of two methods to establish efficacy of disease diagnosis. This work was undertaken by the patient’s care team, and looked only at bacteria in blood samples. It did not involve the analysis or study of relevant material as defined by the Human Tissue Authority and so ethical approval was not required. This is in accordance with the revised guidance in the Governance Arrangements for Research Ethics Committees (GAfREC) that was released in September 2011 and was found to be fully compliant the NHS Research Governance Framework for Health and Social Care (April 2005), and with all other current regulatory requirements.

## Results

A total of 115 patient cases were included in this study. The most common day one Gram stain finding was Gram positive cocci resembling Staphylococci (49/115), followed by Gram negative rods (32/115) (Table 1).

**Table 1:** Results of positive blood culture Gram stains on day one.

Gram Stain Result	Cases (%)
Staphylococci	49 (43)
Gram Negative Rods	32 (28)
Streptococci	15 (13)
Gram Positive Rods	10 (8)
Mixed Gram Stain	7 (6)
Gram Negative Cocci	1 (<1)
Haemolysed Bottle	1 (<1)

### Comparison of culture identification with direct MALDI-ToF identification

Of the 115 cases included, 108 (93.9%) achieved identification of one or more isolates from blood through routine culture and 73 (63.5%) had a pathogen identified with the direct MALDI-ToF identification technique on day one. Of those with a direct identification, 70/73 (95.9%) had a result concordant with the culture result. Three cases had a direct identification that was discordant with the culture result. The discordant results are shown in Table 2.

**Table 2:** Discordant results of conventional culture vs. direct MALDI-ToF identification.

Organisms seen in direct blood culture Gram stain	Organisms identified from growth plate sub-culture	Organisms identified by direct MALDI-ToF
Streptococcus and Yeast	*Candida albicans* and *Granulicatella*	*Staphylococcus epidermidis*
Staphylococcus and Streptococcus	*Enterococcus faecalis* and Mixed coagulase negative staphylococci	*Staphylococcus haemolyticus*
Streptococcus	Mixed coagulase negative staphylococci and Gram positive rod	*Granulicatella adiacens*

Of the 28 cases from which a single Gram negative organism was identified by culture, 26 (89%) had the Gram negative organism identified by MALDI-ToF. Of the 60 cases from which a single Gram positive organism was identified by culture, 32 (53%) had the Gram positive organism identified by MALDI-ToF.

### Overall clinical impact

In 28 of the 115 included cases, early MALDI-ToF identification would have had clinical benefit. When determining the impact of early organism identification each clinical impact category was considered separately. As a result some cases fell into more than one category, for example an early identification may have provided information of source of infection and informed antimicrobial agent choice. The results for each clinical impact category is reported below.

### Determining clinical significance of a positive blood culture

Of the 115 patient cases, 102 episodes of communication between an infection specialist and the primary clinical team were made on day one of the blood culture result. This consisted of a bedside review or telephone consultation where the Gram stain result was relayed, as well as advice regarding investigation, source control, antimicrobial chemotherapy and infection control measures where appropriate. On the basis of clinical judgement, 11 Gram stain results were not communicated to clinical teams on day one. In addition, two patients had died before the blood culture result was available and therefore the result was not conveyed to a clinical team. With the benefit of direct MALDI-ToF identification, a total of 14 additional cases (12%), all coagulase negative staphylococci, could have had the blood culture growth designated as ‘of doubtful clinical significance’ on day one. This would have avoided 14 episodes of communication with clinical teams that did not influence patient care.

### Identifying the source of infection and making recommendations for investigation

Direct MALDI-ToF identification on day one would have indicated a different source of infection or changed the advice regarding investigation of the patient in 11 out of 115 cases (9.6%). Ten of these involved a Gram positive organism (3 cases *Staphylococcus aureus*, 2 cases *Enterococcus faecalis*, and one case of each of the following organisms; *Streptococcus pneumoniae*, *Staphylococcus hominis*, *Micrococcus luteus*, *Streptococcus sanguinis*). The only case including a Gram negative organism involved a blood culture with a day one Gram of Gram positive cocci resembling Staphylococci which cultured only an *Escherichia coli*. The direct MALDI-ToF method correctly identified this blood culture as containing *E*. *coli* on day one.

### Selection of antimicrobial agents

In 11 cases (9.6%), the MALDI-ToF identification of an organism on day one would have been an indication for changing the antimicrobial agent selected on the basis of the Gram stain result. In the nine cases where a Gram positive organism was isolated, five of the patients could have received more narrow-spectrum antimicrobial therapy from day one (Table 3). In the two Gram negative bacteraemia cases, one patient could have received more narrow-spectrum antibiotics from day one. Knowing the identification of an organism on day one would have facilitated five patients receiving appropriate antibiotics 24 hours sooner in their illness (highlighted in bold in Table 3).

**Table 3:** Summary of antimicrobial changes made with organism identification.

Organisms seen in direct blood culture Gram stain day one (number of bottles positive)	Antibiotics recommended on day one	Organisms identified by direct MALDI-ToF on day one	Organisms identified from growth on plate sub-culture day two	Antibiotic change on day two
Staphylococcus (2/2)	Vancomycin	*S*. *aureus*	*S*. *aureus*	Flucloxacillin
**Streptococcus (2/2)**	**Nil**	***E*. *faecalis***	***E*. *faecalis***	**Amoxicillin**
Gram negative rod (1/2)	Meropenem	*Klebsiella pneumoniae*	*K*. *pneumoniae*	Ertapenem
Streptococcus (2/2)	Co-amoxiclav	*S*. *pneumoniae*	*S*. *pneumoniae & S*. *capitis*	Benzylpenicillin
**Staphylococcus (1/2)**	**Flucloxacillin**	***M*. *luteus***	***M*. *luteus***	**Vancomycin**
**Streptococcus (2/2)**	**Ertapenem**	***E*. *faecium***	***E*. *faecium***	**Ertapenem and vancomycin**
**Streptococcus (2/2)**	**Meropenem**	***E*. *faecium***	***E*. *faecium***	**Meropenem and vancomycin**
Gram negative rod (2/2)	Tazocin	*E*. *cloacae*	*E*. *cloacae*	Meropenem
Staphylococcus (2/2)	Tazocin	*S*. *aureus*	*S*. *aureus*	Flucloxacillin
Streptococcus (2/2)	Amoxicillin	*S*. *sanguinis*	*S*. *sanguinis*	Benzylpenicillin
Staphylococcus (2/2)	Vancomycin	*S*. *aureus*	*S*. *aureus*	Flucloxacillin

### Infection prevention and control

No cases had the potential for an earlier infection control intervention as a result of the direct MALDI-ToF identification. During the data collection period there was one case of *Streptococcus pyogenes* bacteraemia. In this particular patient, *S*. *pyogenes* had already been isolated from a knee aspirate and appropriate infection control measures were already in place. As such, there was no indication for any additional infection control intervention in response to the identification of the blood culture isolate.

## Discussion

Results from our study demonstrate that direct identification of organisms in positive blood culture bottles using MALDI-ToF can have a clinical impact in patients with bacteraemia. Day one identification of organisms from positive blood cultures, particularly Staphylococcal species, assists the infection specialist in assessing the clinical significance of a blood culture isolate, allows streamlining of clinical services, and prioritisation of cases where consultation is most likely to impact on patient care. As Martiny *et al* [7] highlighted, this is likely to be of particular help in the paediatric setting where the blood culture contamination rate is high. There is also potential for patients to avoid unnecessary exposure to antimicrobials if clinicians can be assured of the identification of a blood culture isolate. In both respects, use of MALDI-ToF for day one identification can be considered a service quality improvement, the impact of which is experienced by both the infection specialist and the wider hospital community.

Accurately identifying the source of infection is a key component in determining how to manage a patient with bacteraemia. MALDI-ToF identification of a Gram positive organism helps to narrow down the potential source of infection and provide a focus for clinical investigation from day one. Our results suggest this is most often the case for species that appear as Streptococci in a Gram stain, for example the identification of *E*. *faecalis* would prompt abdominal investigations. Equally, establishing that an organism is likely to be a contaminant would allow the infection specialist to confidently forgo further investigation that may be under consideration, for example ruling out a *S*. *aureus* bacteraemia on day one would avoid unnecessary additional imaging and repeat blood cultures. Whilst the benefit of organism identification is apparent for Gram positive organisms, for cases of Gram negative bacteraemia early organism identification is less likely to contribute to source identification or investigation.

Even in the absence of susceptibility testing, organism identification can directly influence the choice of antimicrobial agent. These results concur with a published study by Vlek et al [11] who also demonstrated MALDI-ToF identification improves appropriateness of antibiotic therapy 24 hours after blood culture positivity. From our results, two scenarios can be characterised: 1) The organism identification is that of a common pathogen with a somewhat predictable susceptibility profile. This allows narrow spectrum antibiotics to be advised from day one e.g. *Streptococcus pneumoniae*. 2) The organism identification is that of a less common pathogen suggesting antibiotics with a broader spectrum of activity are required from day one than would typically be given on the Gram stain result alone e.g. *Enterobacter cloacae*. By facilitating the use of more narrow-spectrum antibiotics, the direct MALDI-ToF method can be viewed as contributing positively to antimicrobial stewardship. When considering cases where broader-spectrum antibiotics are indicated, organism identification allows earlier appropriate antimicrobial therapy, which may otherwise be delayed. A similar impact was documented by Clerc *et al* [10] who described MALDI-ToF identification altering antibiotic management in 59.3% of bacteraemias caused by AmpC-producing *Enterobacteriaceae*. Results from our study have demonstrated a potential impact on prescribing that can benefit the individual patient and the wider patient population within a healthcare organisation.

For commonly encountered Gram negatives with a highly variable susceptibility profile, such as *E*. *coli*, organism identification alone is less helpful for informing antimicrobial agent choice. However, advances in the use of MALDI-ToF for generating susceptibility profiles [12–14] suggests that utilisation of the mass spectrometry technology is only set to increase. Rapid detection of organisms with highly resistant susceptibility profiles, such as carbapenemase producing enterobacteriaceae (CPE), would not only facilitate more appropriate antimicrobial choice but also give MALDI-ToF a vital role in hospital based infection prevention and control. Most indications for infection control interventions, such as patient isolation, are dependent upon the antimicrobial susceptibility profile of an organism. If use of direct MALDI-ToF identification can provide this information, earlier implementation of heightened infection control measures will reduce the risk of transmission of these organisms. This would be a valuable tool in the on-going effort to keep our patients safe from healthcare associated infections.

Use of direct MALDI-ToF identification has significant potential, however limitations must also be acknowledged. The proportion of positive blood culture bottles that will not generate a reliable identification limits the clinical impact of direct MALDI-ToF use. In our study we demonstrated a successful MALDI-ToF identification in 53% of cases with single Gram positive organisms, significantly lower than the identification rate of Gram negative organisms (89%). This is consistent with a number of previously published studies that found a significantly better rate of acceptable identification result in Gram negative organisms [3,4,15]. As cases of Gram positive bacteraemia are the scenario in which day one identification currently has the greatest potential benefit, the rate of reliable identification in this group is critical.

Our study design, through necessity, did not allow clinical decision-making as a result of direct MALDI-ToF results. As a result, significant clinical outcomes such as patient mortality and length of stay could not be measured. Instead, potential clinical impacts were assessed as outcomes measures and the authors acknowledge this as a limitation of our study. For this reason, further prospective investigation of the clinical impact of using direct MALDI-ToF identification is warranted and would contribute further to the body of evidence on this topic.

In addition to using MALDI-ToF to generate susceptibility profiles, expanding the type of clinical specimen that can be analysed by mass spectrometry presents opportunities for new diagnostic pathways. MALDI-ToF has been rapidly adopted by hospital-based laboratories, in part due to its ease of use and contribution to improved laboratory workflow. The expanded use of MALDI-ToF has huge potential and few barriers in terms of additional training or demands on laboratory time. As this technology evolves to accurately identify a broader range of organisms and resistance mechanisms, the clinical impact of MALDI-ToF on diagnosis, antimicrobial stewardship and infection control will surely increase.
